# Supplementary material for: KMT2E recruitment by H3 serotonylation regulates neurodevelopmental chromatin dynamics
Source: bioRxiv. 2026 Sep 22:2026.09.20.752984. Preprint. [Version 1] doi: 10.64898/2026.09.20.752984 (PMC13622148; doi:10.64898/2026.09.20.752984)
Supplement: Supplementary file 1 [file NIHPP2026.09.20.752984v1-supplement-2.pdf]

bioRxiv preprint doi: https://doi.org/10.64898/2026.09.20.752984; this version posted September 22, 2026. The copyright holder for this preprint (which was not certified by peer review) is the author/funder, who has granted bioRxiv a license to display the preprint in perpetuity. It is made available under a CC-BY 4.0 International license.

SUPPLEMENTARY MATERIALS

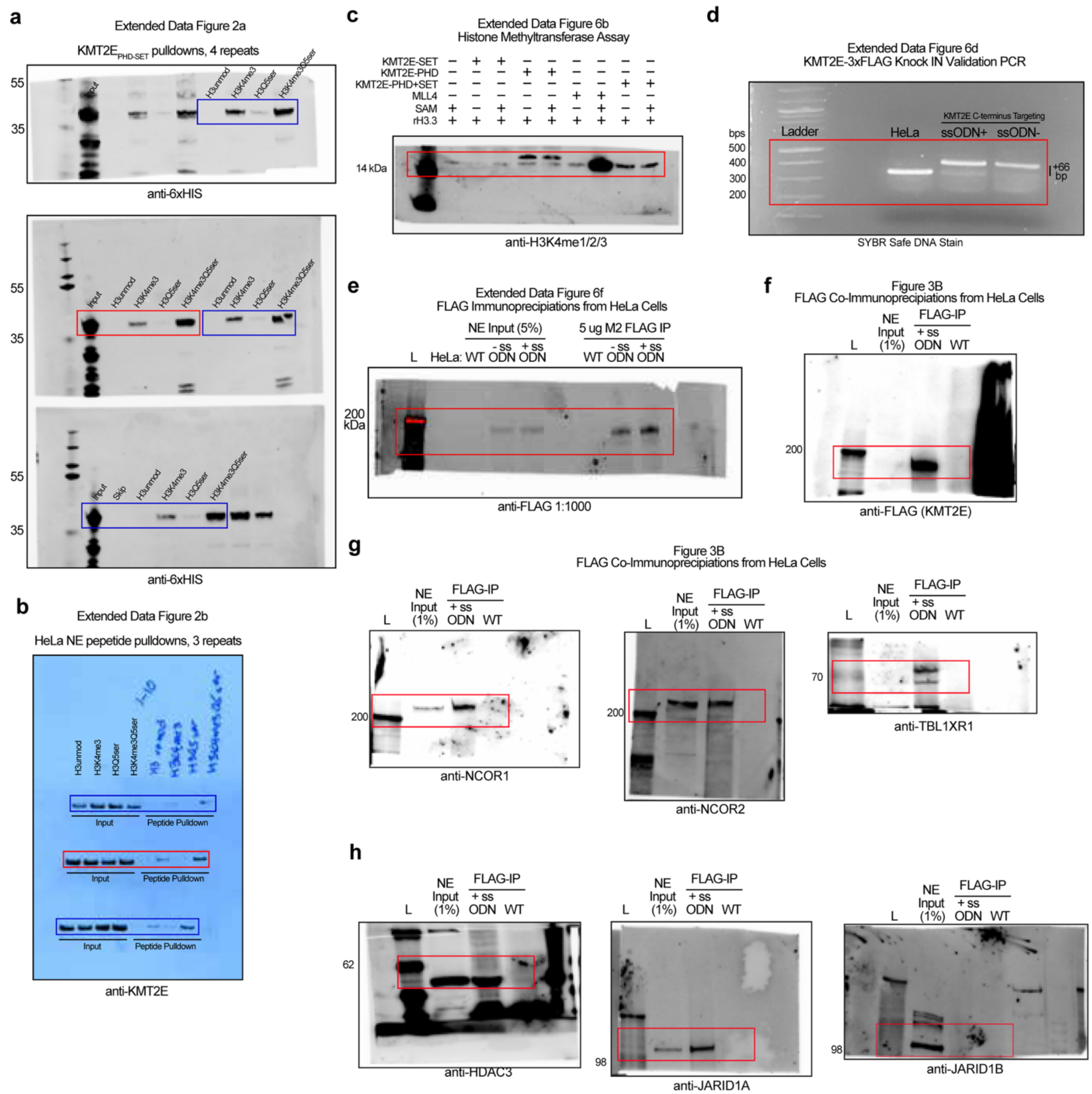

bioRxiv preprint doi: https://doi.org/10.64898/2026.09.20.752984; this version posted September 22, 2026. The copyright holder for this preprint (which was not certified by peer review) is the author/funder, who has granted bioRxiv a license to display the preprint in perpetuity. It is made available under a CC-BY 4.0 International license.

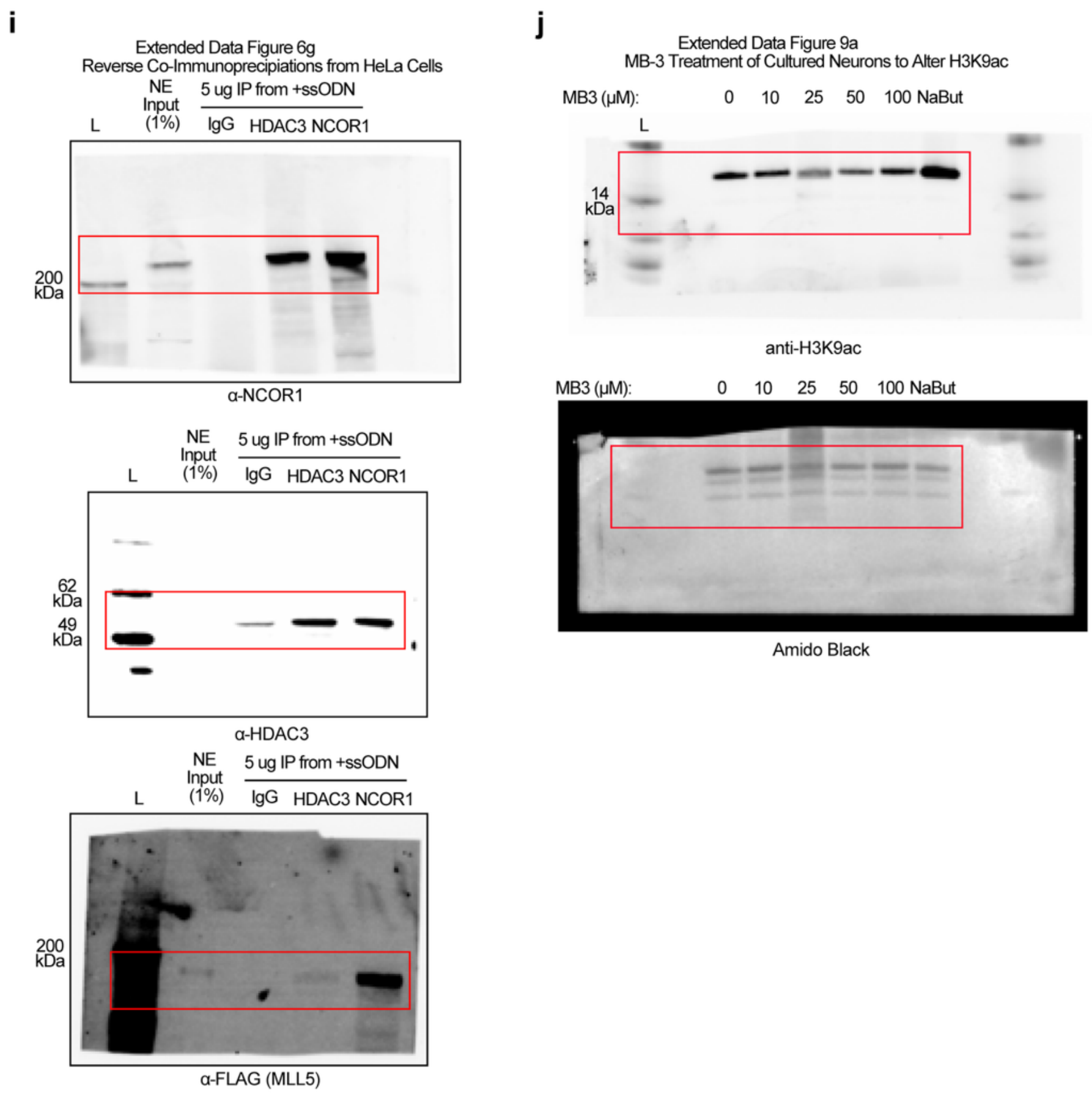

Supplementary Figure 1: Uncropped Immunoblots
Uncropped immunoblots related to (**a**) Extended Data Fig. 2a, (**b**) Extended Data Fig. 2b, (**c**) Extended Data Fig. 6b, (**d**) Extended Data Fig. 6d, (**e**) Extended Data Fig. 6f, (**f-h**) Fig. 3b , (**i**) Extended Data Fig. 2g, (**j**) Extended Data Figure 9a. Red rectangles notate portion of blot included in figures, and blue rectangles notate portion of blots where analysis was performed but were not used as representative images.

Supplementary Data:
Supplementary Table 1: H3K4me3Q5ser broad vs. narrow peaks in E12.5 brain

Supplementary Table 2: DESeq2 table for WT vs. KO cerebellum RNA-seq

Supplementary Table 3: DESeq2 table for WT vs. KO mPFC RNA-seq

Supplementary Table 4: Differential splicing table for WT vs. KO rMATs analysis

Supplementary Table 5: LC-MS/MS analysis from FLAG-KMT2E immunoprecipitation

Supplementary Table 6: KMT2E-bound TSSs in cerebellum from CUT-RUN-sequencing

Supplementary Table 7: KMT2E-bound DEGs in cerebellum from CUT&RUN-sequencing

bioRxiv preprint doi: https://doi.org/10.64898/2026.09.20.752984; this version posted September 22, 2026. The copyright holder for this preprint (which was not certified by peer review) is the author/funder, who has granted bioRxiv a license to display the preprint in perpetuity. It is made available under a CC-BY 4.0 International license.

Supplementary Table 8: DESeq2 table for WT vs. HET from granule neuron RNA-seq

Supplementary Table 9: DESeq2 table for WT vs. KO from granule neuron RNA-seq

Supplementary Table 10: KMT2E peaks in granule neurons from CUT&RUN-sequencing

Supplementary Table 11: DESeq2 table for WT-Veh vs. HET-Veh from granule neuron RNA-seq

Supplementary Table 12: DESeq2 table for HET-MB-3 vs. HET-Veh from granule neuron RNA-seq

Supplementary Table 13: Clusters of WT-Veh vs. HET-Veh DEGs altered by MB-3

Source Data File
